# Relationship between polygenic risk scores and symptom dimensions of schizophrenia and schizotypy in multiplex families with schizophrenia

**DOI:** 10.1192/bjp.2022.179

**Published:** 2023-07

**Authors:** Mohammad Ahangari, Daniel Bustamante, Robert Kirkpatrick, Tan-Hoang Nguyen, Brian C. Verrelli, Ayman Fanous, Kenneth S. Kendler, Bradley T. Webb, Silviu-Alin Bacanu, Brien P. Riley

**Affiliations:** Virginia Institute for Psychiatric and Behavioral Genetics, Virginia Commonwealth University, USA; and Integrative Life Sciences PhD Program, Virginia Commonwealth University, USA; Virginia Institute for Psychiatric and Behavioral Genetics, Virginia Commonwealth University, USA; and Department of Psychiatry, Virginia Commonwealth University, USA; Center for Biological Data Science, Virginia Commonwealth University, USA; Department of Psychiatry, University of Arizona, USA; Virginia Institute for Psychiatric and Behavioral Genetics, Virginia Commonwealth University, USA; Department of Psychiatry, Virginia Commonwealth University, USA; and Department of Human and Molecular Genetics, Virginia Commonwealth University, USA; GenOmics, Bioinformatics, and Translational Research Center, Biostatistics and Epidemiology Division, RTI International, Research Triangle Park, USA

**Keywords:** Schizophrenia, psychotic disorders, genetics, schizotypy, negative symptoms

## Abstract

**Background:**

Psychotic disorders and schizotypal traits aggregate in the relatives of probands with schizophrenia. It is currently unclear how variability in symptom dimensions in schizophrenia probands and their relatives is associated with polygenic liability to psychiatric disorders.

**Aims:**

To investigate whether polygenic risk scores (PRSs) can predict symptom dimensions in members of multiplex families with schizophrenia.

**Method:**

The largest genome-wide data-sets for schizophrenia, bipolar disorder and major depressive disorder were used to construct PRSs in 861 participants from the Irish Study of High-Density Multiplex Schizophrenia Families. Symptom dimensions were derived using the Operational Criteria Checklist for Psychotic Disorders in participants with a history of a psychotic episode, and the Structured Interview for Schizotypy in participants without a history of a psychotic episode. Mixed-effects linear regression models were used to assess the relationship between PRS and symptom dimensions across the psychosis spectrum.

**Results:**

Schizophrenia PRS is significantly associated with the negative/disorganised symptom dimension in participants with a history of a psychotic episode (*P* = 2.31 × 10^−4^) and negative dimension in participants without a history of a psychotic episode (*P* = 1.42 × 10^−3^). Bipolar disorder PRS is significantly associated with the manic symptom dimension in participants with a history of a psychotic episode (*P* = 3.70 × 10^−4^). No association with major depressive disorder PRS was observed.

**Conclusions:**

Polygenic liability to schizophrenia is associated with higher negative/disorganised symptoms in participants with a history of a psychotic episode and negative symptoms in participants without a history of a psychotic episode in multiplex families with schizophrenia. These results provide genetic evidence in support of the spectrum model of schizophrenia, and support the view that negative and disorganised symptoms may have greater genetic basis than positive symptoms, making them better indices of familial liability to schizophrenia.

## Background

Schizophrenia (SCZ) is a clinically heterogeneous psychiatric disorder with a population prevalence of ~1%.^1^ In the past decade, genome-wide association studies (GWAS), copy number variation studies and rare variant studies have significantly improved our understanding of the genetic basis of SCZ.^2,3^ As a result of the heterogeneous manifestation of SCZ symptoms, studies have attempted to capture this clinical heterogeneity in terms of symptom dimensions derived from factor analyses. Although these derived dimensions vary across studies, they often result in positive, negative/disorganised and affective dimensions.^4^

## Polygenic risk scores (PRSs) and schizophrenia symptom dimensions

In recent years, the relationship between aggregate common risk variation indexed by PRSs and clinical dimensions of SCZ has garnered much attention. Early studies using the first wave of Psychiatric Genomics Consortium Schizophrenia (PGC)-SCZ GWAS found no association between SCZ PRS and symptom dimensions, likely because of the smaller sample size and lower power of PGC1-SCZ GWAS.^5^ Recent analyses using the second wave of PGC-SCZ GWAS have found significant associations between SCZ PRS and negative and disorganised dimensions, suggesting that polygenic liability to SCZ can explain part of the variance in negative and disorganised symptoms.^6,7^ Most recently, Smigielski and colleagues^8^ showed that PGC3-SCZ PRS is also significantly associated with dimensions from the Positive and Negative Syndrome Scale.

## PRSs and schizotypy symptom dimensions

In addition to the heterogeneous manifestation of SCZ, some relatives of probands with SCZ, although never having a psychotic episode, exhibit clinical features that closely resemble those observed in their ill relatives.^9^ In the Danish Adoption Study of SCZ, these symptoms and signs differentiated the relatives of probands with SCZ from controls and were later combined into the classification of schizotypal personality disorder in the DSM-III.^10^ Since then, considerable evidence from family, adoption and twin studies suggests that schizotypal traits aggregate in relatives of probands with SCZ.^11^ Although earlier studies have linked specific genes to schizotypal traits,^12^ the relationship between SCZ PRS and schizotypal traits has not been fully established.^13^ Recently, subclinical phenotypes such as psychotic-like experiences have been proposed to be used as proxies to capture subclinical liability to psychosis. For example, Legge and colleagues^14^ analysed the UK Biobank cohort and showed that psychotic-like experiences have pleiotropic association with polygenic liability to SCZ and other psychiatric and neurodevelopmental disorders. However, these findings indicate that unlike schizotypal traits, which significantly aggregate in the relatives of probands with SCZ, psychotic-like experiences are not specific to SCZ. This is further strengthened by studies showing that rates of psychotic-like experiences do not differ significantly between relatives and non-relatives of patients with SCZ in clinically ascertained samples.^15^

## Aims

Participants ascertained from multiplex families with SCZ represent the upper bounds of SCZ risk in the population, and a major question is the extent to which symptom severity in SCZ can be attributed to genetic differences among participants. We have previously shown that members of the Irish Study of High-Density Schizophrenia Families (ISHDSF)^16^ have an increased PRS for SCZ, bipolar disorder (BIP) and major depressive disorder (MDD) compared with population controls.^17,18^ In this study, we sought to examine the differential relationship between PRSs for these three major psychiatric disorders, and quantitative measurement of symptom severity in the ISHDSF sample.

We hypothesise that by using a well-ascertained sample of multiplex families with SCZ, we will be able to identify associations between SCZ PRSs and core SCZ symptom dimensions in participants with a history of a psychotic episode (psychotic episode group), while also maximising power to uncover specific associations between SCZ PRSs and schizotypal dimensions in participants without a history of a psychotic episode (non-psychotic episode group) in the families. To the best of our knowledge, this study is the first that aims to establish a relationship between SCZ PRS and symptom dimensions in people with SCZ and their relatives across the extended psychosis spectrum.

## Method

### ISHDSF

Fieldwork for the ISHDSF sample was conducted between 1987 and 1992 from public psychiatric hospitals in the Republic of Ireland and Northern Ireland. Selection criteria were two or more first-degree relatives meeting the DSM-III-R criteria for SCZ or poor-outcome schizoaffective disorder, with all four grandparents born in Ireland or the UK. Relatives of the probands suspected of having psychotic illness were interviewed by trained psychiatrists. Trained social workers interviewed other relatives. To avoid bias and detect possible diagnostic errors, an independent review of all diagnostic information was made masked to family assignments by two trained psychiatrists, each making up to three best estimate DSM-III-R diagnoses, with a high agreement (weighted kappa = 0.94 +/− 0.05).

The diagnostic schema of the ISHDSF sample follows a concentric pattern ranked by the degree to which they reflect the core and the periphery of the psychosis spectrum. This includes four case definitions as follows:

- narrow spectrum;
- intermediate spectrum;
- broad spectrum; and
- very broad spectrum.

The sample also includes unaffected family members with no diagnosis of any psychiatric illness. More information is provided elsewhere^16^ and in the Supplementary Appendix S1 under the ISHDSF section, available at https://doi.org/10.1192/bjp.2022.179.

All participants provided informed consent to participate in the study procedures. All procedures contributing to the sample collection comply with the ethical standards of the relevant national and institutional committees on human experimentation and with the Helsinki Declaration of 1975, as revised in 2008. Procedures were approved by St. James Hospital/Adelaide and Meath Hospital – National Children's Hospital (SNJH-AMNCH) Research Ethics Committee with approval number 2009/09/04, Scotland A Research Ethics Committee with approval number 11/SS/0041 and Virginia Commonwealth University Institutional Review Board with approval number HM12497.

### Symptom dimensions in participants with a history of a psychotic episode

For participants with a lifetime occurrence of a psychotic episode (*n* = 539) (psychotic episode group), the Operational Criteria Checklist for Psychotic Disorders (OPCRIT)^19^ was completed based on the review of detailed hospital records and interviews to assess the symptom dimensions. A full description of the factor analysis of OPCRIT in the ISHDSF sample is provided elsewhere.^20^ Briefly, 55 of the 75 items of the OPCRIT were entered into the factor analysis. These items were selected because they represent signs and symptoms rather than the course of illness.

Five factors were derived and factor-derived scores were generated. These five factors were identified as (a) negative/disorganised, (b) hallucinations, (c) delusions, (d) manic symptoms, (e) depressive symptoms. The full list of items and their loadings are provided in Supplementary Table 1.

### Symptom dimensions in participants without a history of a psychotic episode

For participants without a lifetime occurrence of a psychotic episode (*n* = 322) (non-psychotic episode group), the Structured Interview for Schizotypy (SIS)^21^ was used to assess schizotypal signs and symptoms across the psychosis spectrum. The items used included the DSM-III-R major signs and symptoms of schizotypal personality disorder. SIS was originally developed from family studies of SCZ in the west of Ireland.^22^ It includes signs and symptoms that are specific to schizotypy, with a contextual assessment of the pathological nature of symptoms that can significantly discriminate the relatives of probands with SCZ from that of controls. Based on our hypothesis that schizotypy captures a continuous measure of liability to SCZ in relatives of probands with SCZ, the SIS was also conducted in unaffected relatives to capture the symptom dimensions on the extended psychosis spectrum.

### Factor analysis of SIS in the non-psychotic episode group

Exploratory (EFA) and confirmatory (CFA) factor analyses were conducted to determine and verify the least number of factors explaining the maximum amount of variance in the SIS data, using the R packages psych (R package version 2.2.9, 2022, Northwestern University, Evanston, Illinois, USA; https://CRAN.R-project.org/package=psych) and OpenMx.^23^ The maximum likelihood polychoric correlations were estimated using the R package polycor (R package version 0.8–1, 2022, McMaster University, Hamilton, Ontario, USA; https://CRAN.R-project.org/package=polycor) to obtain the eigenvalues and eigenvectors, using a minimum eigenvalue of 1 as the cut-off. EFA with two and three factors were conducted using an oblique and orthogonal rotation. The minimum cut-off for each indicator factor loadings was set at ≥0.3, considering only the highest loading if one indicator loaded into more than one factor. Two independent fits of CFA with two and three factors were implemented to corroborate the factor structure, and factor scores were generated using the maximum likelihood method.

### Genotyping and imputation

We genotyped 830 participants on the Illumina 610-Quad Array at the Illumina genotyping site in the USA, and an additional 175 participants were later genotyped on the Infinium psychArray V.1.13 Array (psychChip) at Mount Sinai. Exclusion criteria for samples were a call rate of <95%, >1 Mendelian error and a difference between reported and genotypic gender. Exclusion criteria for single nucleotide polymorphisms (SNPs) were minor allele frequency <1%, call rate <98% and *P* < 0.0001 for deviation from Hardy–Weinberg expectation. The final sample included 861 individuals from 253 families for whom OPCRIT or SIS data were available.

Genotypes passing quality control were phased and imputed to the Haplotype Reference Consortium (HRC) reference panel on the Michigan Imputation Server.^24^ After imputation and quality control, 9 298 012 SNPs on the Illumina Array, and 11 081 999 SNPs on the psychChip with minor allele frequency (MAF) > 1% remained for analysis. After merging, 9 008 825 SNPs were shared across the two arrays. The imputation quality score for the shared SNPs that went into PRS construction and downstream analyses were high (*r*^2^ > 0.96). More information is provided in the Supplementary Appendix S1 under the imputation quality section, and Supplementary Tables 2 and 3.

### Polygenic risk score construction

We constructed PRS using the PRS-CS method that shows substantial improvement over the traditional clumping method.^25^ To avoid upward bias in SCZ PRS estimations, leave-*N*-out SCZ summary statistics were acquired from the PGC by excluding the Irish cohort. Preparation of the summary statistics for leave-*N*-out PGC3-SCZ (*n* = 156 509), PGC3-BIP (*n* = 413 466) and PGC2-UKB-MDD meta-analysis (*n* = 500 199) followed standard quality control by excluding variants with MAF < 1%, imputation quality score of <0.9, and removing all strand ambiguous variants and indels. PRS-CS limits SNPs for PRS construction to approximately 1.2 million high-quality HapMap3 variants, and uses linkage disequilibrium information from the 1000 Genomes European Phase 3 sample^26^ to estimate the posterior effect sizes of each SNP. The constructed PRS were normalised using *Z*-score standardisation for downstream analyses.

### Statistical analyses

To account for the family structure in the sample, the genomic relationship matrix was constructed using LDAK,^27^ and included in the mixed models as a random effect. Sex, genotyping platform, genotyping sites, age at interview and the top 10 principal components were also included as additional covariates. More information on the principal component analysis, covariates and handling of possible batch and site effects are provided in the Supplementary Appendix S1 under the principal component analysis section, and Supplementary Figs 1–3.

Association analyses were carried out using a two-step approach. First, mixed-effects linear regression analyses were performed using lmekin in R (R-package version 2.2–18.1, 2022, Mayo Clinic, Rochester, Minnesota, USA; https://CRAN.R-project.org/package=coxme). Given that the floor effect in some of the symptom dimensions could violate the assumptions of a linear regression,^28^ we also conducted mixed-effects quantile-regression analyses on dimensions that showed significant association with PRSs using the qrLMM package in R.^29^ Quantile regression is an extension of linear regression that estimates the effects at different locations in the distribution without the need to have normality assumptions met. Whereas linear regression uses the mean as a measure of centrality, quantile regression looks at a number of pre-defined quantiles of the data across the distribution. In situations with an abundance of zero responses such as symptom measurements, the quantile-regression method provides a more accurate estimation of the centrality of the data at different locations in the distribution of symptom scores. We used three tau (τ) values (0.25, 0.5, 0.75), representing 25% (quantile (Q)1), 50% (Q2), and 75% (Q3) of the symptom severity that fall below the corresponding points in the distribution of symptom severity, respectively. The nominal significance for all analyses was set at *P* < 0.05 and the *p*-values were adjusted for multiple testing using the Holm method. More information is provided in Supplementary Figs 4 and 5.

## Results

### Participants

**Fig. 1:**
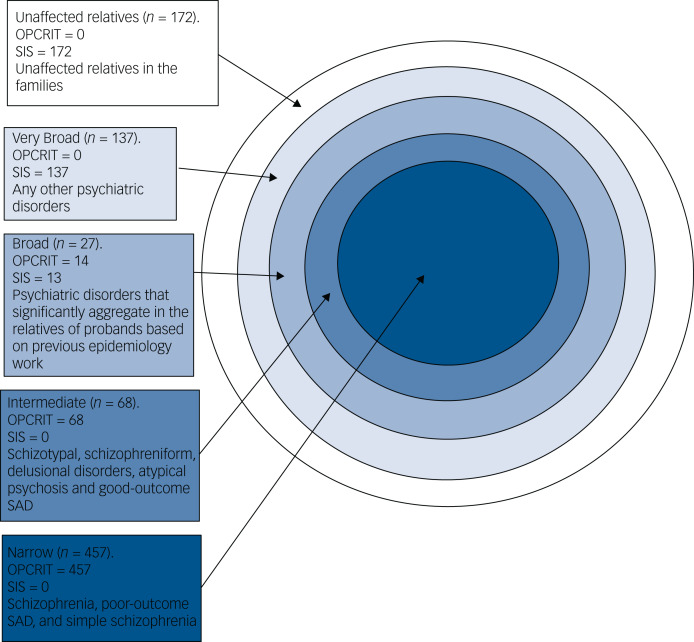
Concentric diagnostic hierarchy of the Irish Study of High-Density Schizophrenia Families (ISHDSF) sample reflecting the core versus periphery of the psychosis spectrum. The diagnostic schema contains four case definitions reflecting the schizophrenia spectrum: (a) narrow spectrum in dark blue, (b) intermediate spectrum in mid-blue, (c) broad spectrum in sky blue, and (d) very broad spectrum in light blue. Additionally, the sample also includes unaffected relatives in the families. Note that the numbers shown in each category reflects those with genotype and symptom-level information available, whereas the full sample includes a larger set of participants. SAD, schizoaffective disorder; OPCRIT, Operational Criteria Checklist for Psychotic Disorders; SIS, Structured Interview for Schizotypy.

Figure 1 provides a visual representation of the diagnostic schema of the ISHDSF sample.

- Participants in the narrow spectrum represent the participants with SCZ or poor-outcome schizoaffective disorder (SAD).
- Participants in in intermediate spectrum represent individuals with diagnoses of other psychotic disorders in the families. Symptom severity in these two spectrums were measured using OPCRIT.
- Broad spectrum includes participants with a diagnosis of a psychiatric disorder that significantly aggregate in the relatives of probands with SCZ. Symptom severity for these individuals were measured using OPCRIT or SIS, depending on whether an individual had a history of a psychotic episode.
- Very broad spectrum includes any other psychiatric disorder present in the families. Symptom severity for these individuals, and unaffected relatives were measured using SIS.

The full list of the psychiatric diagnoses in each diagnostic spectrum is provided in Supplementary Tables 4 and 5.

### Factor analysis of schizotypy symptoms in the non-psychotic episode group

**Fig. 2:**
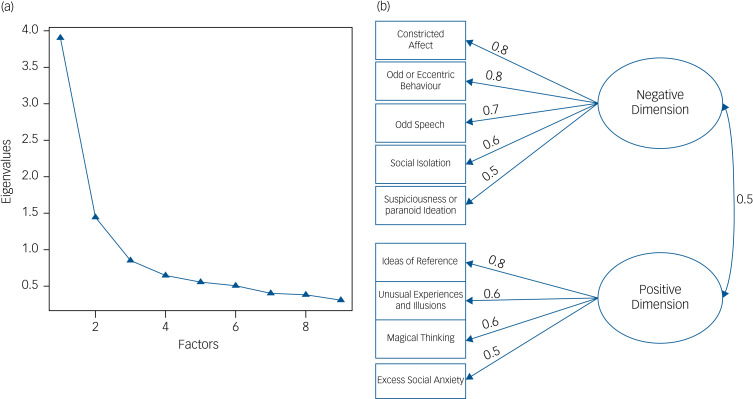
Factor analysis of schizotypy symptoms based on SIS data in members of the Irish Study of High-Density Schizophrenia Families (ISHDSF) sample without a history of a psychotic episode (*n* = 322). (a) Scree plot in exploratory factor analysis of nine major signs and symptoms of schizotypy in participants without a history of a psychotic illness. (b) Path diagram representing the factor loadings of schizotypal signs and symptoms on the two factors representing positive and negative schizotypy. SIS, Structured Interview for Schizotypy.

Two eigenvalues were above the minimum cut-off value of 1, suggesting the factor solution of retaining two factors (Fig. 2(a)). The EFA two-factor model fit under an oblique rotation explained the same cumulative variance (0.49) as the two-factor orthogonal rotation, with similar ranges for their factor loadings (0.5–0.8; Fig. 2(b)). An oblique rotation was selected to allow for correlation (0.51) between the two factors. The CFA models supported the two-factor solution (three-factor: −*2lnL* = 4842.22, Akaike Information Criterion (AIC) = 4920.22; 2-factor: −*2lnL* = 4809.67, AIC = 4883.67, Δ*χ^2^* (2) = −32.55, *P* = 1).

The two schizotypy dimensions were discernible as positive and negative dimensions of schizotypy (Fig. 2(b)). More information on the model fit is provided in Supplementary Table 6.

### Association of polygenic risks with symptom dimensions in the psychotic episode group

**Fig. 3:**
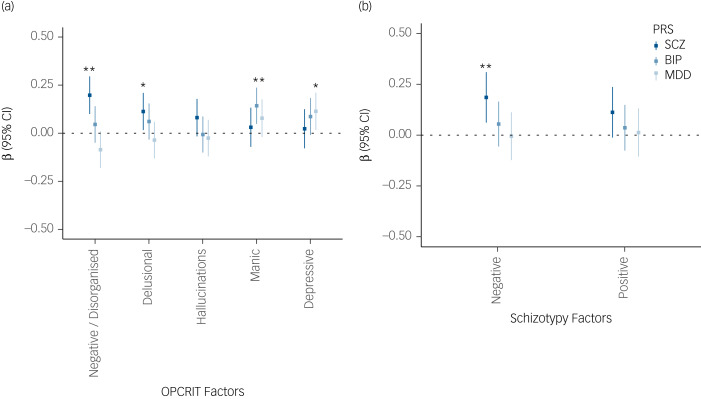
Association of schizophrenia (SCZ), bipoloar disorder (BIP) and major depressive disorder (MDD) polygenic risk scores (PRSs) with OPCRIT and schizotypy symptom dimensions in the Irish Study of High-Density Schizophrenia Families (ISHDSF) sample. Five symptom dimensions were derived from OPCRIT factor analysis in participants with a history of a psychotic episode. Two-factor dimensions were derived from SIS schizotypy factor analysis in participants without a history of a psychotic episode. Error bars represent 95% confidence intervals (95% CI) of the β value. The dotted line at zero represents a null model. Values more than 0 indicate increased risk, whereas values less than 0 indicate reduced risk. *X*-axis shows symptom dimensions. *Y*-axis shows the β value. OPCRIT, Operational Criteria Checklist for Psychotic Disorders; SIS, Structured Interview for Schizotypy. **Significant after multiple testing correction (*P* <0.01) . *Nominally significant at *P* < 0.05. Genomic relationship matrix, gender, genotyping platform, genotyping site, age at interview and the top 10 principal components were included as covariates in the regression analyses.

Figure 3(a) shows the results for the associations between PRS and OPCRIT symptom dimensions in the psychotic episode group. SCZ PRS was found to be a significant predictor of the negative/disorganised symptom dimension (β = 0.198; 95% CI 0.099–0.305; *P* = 2.31 × 10^−4^). BIP PRS was found to be a significant predictor of the manic symptom dimension (β = 0.181; 95% CI 0.061–0.241; *P* = 3.70 × 10^−4^). SCZ and MDD PRSs also showed suggestive associations with delusional and depressive symptoms respectively, but they did not survive multiple testing correction.

To narrow down the association between SCZ PRS and the negative/disorganised dimension, two additional mixed-effects linear regression analyses were carried out by separating the symptoms into two groups representing negative and disorganised symptoms separately. A significant association was observed for both negative only (β = 0.188; 95% CI 0.081–0.291; *P* = 9.10 × 10^−3^) and disorganised only (β = 0.199; 95% CI 0.091–0.312; *P* = 1.41 × 10^−5^) symptoms. More information is provided in Supplementary Table 7.

### Association of polygenic risks with symptom dimensions in the non-psychotic episode group

Figure 3(b) shows the results for the association of polygenic risks with SIS dimensions in the non-psychotic episode group. SCZ PRS was found to be a significant predictor of the negative symptom dimension (β = 0.186; 95% CI 0.080–0.0.289; *P* = 1.42 × 10^−3^), and no significant association was observed with the positive symptom dimension. Additionally, BIP and MDD PRS showed no association with SIS dimensions. Full results are provided in Supplementary Table 7.

### Quantile-regression analysis of the significant associations

**Fig. 4:**
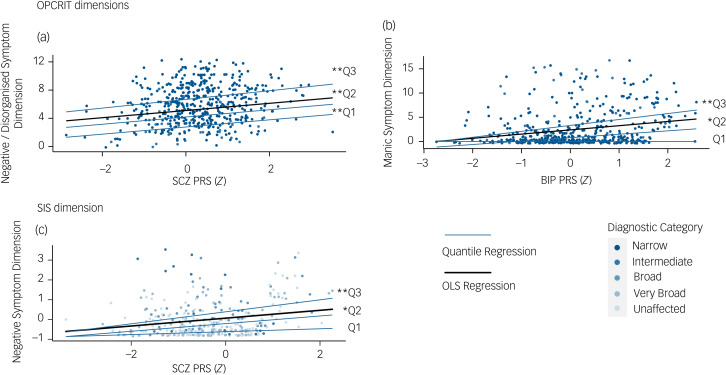
Follow-up quantile-regression analysis of symptom dimensions that showed significant association with polygenic risks. (a) Association of schizophrenia (SCZ) polygenic risk scores (PRSs) with OPCRIT negative/disorganised symptom dimension. (b) Association of bipolar disorder (BIP) PRSs with OPCRIT manic symptom dimension. (c) Association of SCZ PRSs with SIS schizotypy negative symptom dimension. Three quantiles of symptom score distributions were tested (quantile (Q)1 = 0.25, Q2 = 0.50 and Q3 = 0.75), corresponding to the first, second and third quantile of symptom score distributions. The three quantiles are represented with blue lines, while the ordinary least square (OLS) is represented with a black line in each plot. *X*-axis represents normalised PRS. *Y*-axis represents symptom dimension scores. OCPRIT, Operational Criteria Checklist for Psychotic Disorders; SIS, Structured Interview for Schizotypy. **Significant after multiple testing correction (*P*<0.01). *Nominally significant at *P* < 0.05. Genomic relationship matrix, gender, genotyping platform, genotyping site, age at interview and the top 10 principal components were included as covariates in the regression analyses.

Figure 4 shows the follow-up mixed-effects quantile-regression analyses for the three dimensions that showed significant association with the polygenic risks. SCZ PRS is significantly associated with the negative/disorganised symptom dimension in the psychotic episode group at first (*t* = 2.47, *P* = 8.16 × 10^−3^), second (*t* = 2.33 *P* = 1.14 × 10^−2^) and third (*t* = 2.55 *P* = 6.76 × 10^−4^) quantiles of symptom severity. In contrast, SCZ PRS is significantly associated with negative symptom dimension in the non-psychotic episode group only at the third quantile (*t* = 3.29 *P* = 6.3 × 10^−4^), and a suggestive association was also observed at the second quantile. Similarly, BIP PRS is also significantly associated with manic symptoms in the psychotic episode group only at the third quantile (*t* = 3.14 *P* = 5.0 × 10^−3^). Full quantile-regression results are reported in Supplementary Table 8.

## Discussion

In this study, we investigated the relationship between PRS for three major psychiatric disorders and symptom dimensions in multiplex families with SCZ. Our results indicate that polygenic liability to SCZ is significantly associated with increased negative/disorganised symptoms in individuals with a history of a psychotic episode, and negative symptoms in individuals without a history of a psychotic episode across the psychosis spectrum. These findings suggest that polygenic liability to negative and disorganised symptoms appear to be specific to SCZ, as no significant association between these core SCZ symptoms and BIP or MDD PRS were observed.

### Factor structure of schizotypy and its association with SCZ PRS

Examination of the scree plot of schizotypy factor structure suggested that a two-factor solution fit the data best. The symptoms and signs included in each dimension were consistent with the observation that schizotypal traits are generally divided into positive and negative dimensions.^30^ Although the use of self-report questionnaires in combination with interview-based measures is likely to provide a more comprehensive assessment of schizotypy, previous work in another family sample from Ireland suggests that interview-based scales have significantly greater predictive power than self-report measures, in particular for negative symptoms.^31^ This observation could be because of the notion that self-report measures may be inherently limited in their ability to assess signs and symptoms that are difficult to assess in self-reports. For example, if an individual has little insight into their guardedness in answering the questions, asking them to describe these characteristics in a self-report questionnaire may be ineffective. We attribute our ability to detect a significant association between SCZ PRS and the negative dimension of schizotypy to the increased power of PGC3-SCZ-derived PRSs, and the use of interview-based measurement of schizotypy. It is also possible that our use of a family-based sample with a high incidence of psychotic disorders, instead of a population-based cohort, also contributed to our ability to detect a significant association. We further note that the association of SCZ PRS with only the negative dimension of schizotypy is in agreement with previous epidemiological findings that show familial predisposition to SCZ in the relatives of probands without a history of a psychotic episode is likely to be better indexed by the negative symptoms.^9^

### Relationship between PRS and symptom dimensions across the psychosis spectrum

Our findings on the association between PRS and symptom dimensions in multiplex families with SCZ provide new insights into the relationship between polygenic liability to SCZ and symptom dimensions across the psychosis spectrum. Previous studies have addressed the existence of a single continuum of liability for SCZ and schizotypy at the phenotypic level by showing that negative symptoms in probands with psychosis were correlated with negative schizotypy symptoms in their relatives without psychosis.^11^ Our results further show that polygenic liability to SCZ is also significantly associated with the negative/disorganised symptom dimension in participants with a history of a psychotic episode, and the negative symptom dimension in their relatives without a history of a psychotic episode in multiplex families. Familial aggregation of negative symptoms has been reported in several studies including the Danish Adoption Study of SCZ,^10^ the Roscommon Family Study of SCZ^22^ and Maudsley Twin Studies of SCZ.^32^ These findings are further reinforced by PRS examinations that show strong polygenic associations with negative symptoms,^6,7^ whereas other studies have also reported polygenic associations with disorganised symptoms in SCZ.^33^ Thus, our findings provide genetic evidence in support of previous epidemiological findings that negative and disorganised symptoms are likely to have a greater familial basis than positive symptoms,^34^ making them better indices of the familial liability to SCZ across the psychosis spectrum. This is further supported by studies that suggest the correlation between negative SCZ and schizotypal symptoms appears higher than the correlation between positive SCZ and schizotypal symptoms.^9^

### Factor structure of SCZ in the ISHDSF sample

We note that the factor structure of OPCRIT in our sample differs slightly from other factor analyses of SCZ.^4^ Although hallucinations and delusions often load on a single factor called positive symptoms, they loaded on two distinct factors in our study. However, these results are supported by neurological studies that show aetiological discontinuities between hallucinations and delusions.^35^ Perhaps more importantly, negative and disorganised symptoms loaded on the same factor in our study instead of forming two distinct factors. Although we acknowledge this as a potential limitation in our study, we note that this factor structure is consistent with our previous factor analysis in this sample using the Major Symptoms of Schizophrenia Scale,^36^ as well as factor structures in other studies.^6,33^ To address this further, we narrowed down the loadings from the negative/disorganised factor into ‘negative only’ and ‘disorganised only’ symptoms and showed that although both negative and disorganised symptoms were still independently associated with SCZ PRS, this association appears stronger with the disorganised symptoms. This result is in agreement with the observation in another study that suggests although both negative and disorganised symptoms show a strong familial basis, disorganised symptoms are likely to have a more direct association with polygenic liability to SCZ.^33^

### Polygenic evidence for the spectrum model of SCZ

Our results also provide genetic evidence in support of the spectrum model of SCZ at the symptom level. Previous studies on symptom dimensions of SCZ have largely focused on sporadic cases with SCZ.^5,8,13^ In contrast, we utilised a well-ascertained sample of families with SCZ, with detailed interview-based symptom information to provide a full assessment of the relationship between polygenic liability to major psychiatric disorders and symptom dimensions across the psychosis spectrum. Although we observed a significant association between SCZ PRS and the negative/disorganised dimension in the psychotic episode group, and the negative dimension in non-psychotic episode group, no significant association between BIP or MDD polygenic risks and these core SCZ symptom dimensions were observed. This result suggests that, unlike SCZ PRS, polygenic liability to BIP or MDD lacks specificity for core SCZ symptom dimensions, providing genetic evidence in support of the continuum model of SCZ at the symptom level.

### Implications

The long-term prognosis of SCZ depends on the severity of negative symptoms, and a major question about the clinical heterogeneity of SCZ is the extent to which these clinical differences are attributable to genetic differences. Our findings suggest that polygenic liability to SCZ is associated with increased negative/disorganised symptoms in individuals with a history of a psychotic episode and negative symptoms in individuals without with a history of a psychotic episode from multiplex families with SCZ. We further showed that in agreement with previous work, polygenic liability to SCZ appears to be more strongly associated with disorganised symptoms, and the quantile-regression analyses suggest that the association between SCZ PRS and negative schizotypy in participants without with a history of a psychotic episode appears to be strongest at the highest level of symptom severity. Together, these findings across the extended psychosis spectrum provide genetic evidence for the spectrum model of SCZ at symptom level, and corroborates previous epidemiological findings that show negative and disorganised symptoms are likely to have a greater genetic basis than positive symptoms, resulting in better indices of familial liability to SCZ.

### Limitations

The analyses presented here should be interpreted in the context of some limitations. First, the number of participants in this sample is modest. Therefore, future studies should replicate these findings in larger family samples. However, to the best of our knowledge, this is the largest family study to date that establishes a link between SCZ PRS and negative/disorganised symptoms across the psychosis spectrum. Second, the factor structure of SCZ symptoms in our sample differs slightly from other studies. These differences could be attributable to instruments, sample ascertainment, phase of illness or the rotation used for determining the factors.

Third, given that no follow-up assessment on the ISHDSF sample was conducted, we cannot conclusively rule out the possibility that the association between SCZ PRS and negative schizotypy dimension could be driven by some unaffected relatives who may have developed SCZ later in life. However, we note that of the 172 unaffected relatives, only 11 are in the risk age group for developing SCZ (3 males between 18 to 25 years and 8 females between 25 to 35 years), suggesting that this is an unlikely source of bias.

Fourth, negative and disorganised symptoms are associated with cognitive deficits in SCZ. Given that no cognitive measurements were available we also cannot rule out the possibility that the association between SCZ PRS and negative/disorganised symptoms in participants with a history of a psychotic episode might be driven by cognitive deficits. Fifth, PRS predictions are currently constrained to individuals of European ancestry. Thus, as sophisticated cross-ancestry PRS methods become available, these findings should be replicated in people with ethnically and geographically diverse backgrounds. Furthermore, as current PRS methods exclude rare and structural variants, some potentially relevant rare and structural variations were omitted. Finally, we did not consider the role of neuroleptics in ameliorating the symptom severity in the participants.

## Data Availability

The scripts used for conducting this study are available upon request from the corresponding author.

## Supplementary material

For supplementary material accompanying this paper visit https://doi.org/10.1192/bjp.2022.179. click here to view supplementary material
