# Supplementary material for: Relationship between polygenic risk scores and symptom dimensions of schizophrenia and schizotypy in multiplex families with schizophrenia
Source: Br J Psychiatry. 2023 Jul;223(1):301–8. doi: 10.1192/bjp.2022.179 (PMC10258219; doi:10.1192/bjp.2022.179)
Supplement: Supplementary file 1 [file bjpsup.zip › S0007125022001799sup002.docx]

**Supplementary Materials for:**

**The relationship between polygenic risk scores and symptom dimensions of schizophrenia and schizotypy in multiplex schizophrenia families**

Ahangari et al (2022)

Supplementary Appendix 1: 2-6

Supplementary Tables: 7-12

Supplementary Figures: 13-17

References: 18

**Supplementary Appendix 1:**

**Irish Study of High-Density Multiplex Schizophrenia Families (ISHDSF)**

The ISHDSF sample was collected between 1987 and 1992 in the Republic of Ireland and Northern Ireland (1) across 39 psychiatric facilities covering over 90% of the population of the island of Ireland. The inclusion criteria of the sample required at least two first-degree relatives meeting the DSM-III-R criteria for schizophrenia or poor-outcome schizoaffective disorder. The full phenotypic sample contains 277 pedigrees and 1,770 individuals while the full genotyped sample includes 257 pedigrees and 1,005 individuals. Of these 1,005 individuals, 861 have symptom level information available and were used in the current study. Compared to first-degree relatives of epidemiologically sampled schizophrenia cases, first-degree relatives of individuals in the ISHDSF sample have a similar risk for schizotypal personality disorder.

The affection status in the ISHDSF sample was is defined by the degree to which they reflect the core versus periphery of the psychosis spectrum: 1) Narrow spectrum, including schizophrenia, poor-outcome schizoaffective disorder, and simple schizophrenia, 2) Intermediate spectrum, including good-outcome schizoaffective disorder, schizotypal personality disorder, schizophreniform disorder, delusional disorder, and psychosis not otherwise specified, 3) Broad spectrum, including psychotic affective illness, paranoid, avoidant, and schizoid personality disorders, and other disorders that significantly aggregate in the relatives of probands based on previous epidemiological work in Ireland (2), 4) Very broad spectrum, which adds any other psychiatric illness present in the families. The sample also includes unaffected family members with no diagnosis of any psychiatric illness. Figure 1 in the main manuscript provides a visual representation of the diagnostic schema of the sample.

**Imputation Quality Control**

We used the following Michigan Imputation Server (3) to impute the genotypes and perform the initial quality control (QC) on them:

First, chunks of 20 Mb were created from the uploaded genotypes. Then, on each chunk, the followings were checked:

1. Number of valid variants in the chunk. A variant was considered to be valid when it is included in the reference panel.
2. Number of variants found in the reference panel for the chunk, where at least 50% of the variants must be included in the reference panel.
3. Sample call rate for the chunk, where at least 50% of the variants must be called for each sample.

Chunks were excluded if number of variants < 3, overlap is < 50% and sample call rate is < 50%.

Second, the followings were checked on the variant level:

1. Only A, C, G, T alleles are allowed
2. Alternate allele frequency (AF) is calculated and all markers with AF > 0.5 are flagged.
3. SNP call rate is calculated
4. Chi square for each variant is calculated by using reference panel vs study data.
5. Allele switches are determined by comparing reference and alternate allele of the reference panel vs study data with A/T and C/G variants ignored.
6. After removing possible allele switches, strand flips are determined by flipping and comparing reference and alternate alleles from the reference panel vs study data.
7. Determine both allele switches and strand flips by combining steps 5 and 6.

Variants are excluded if alleles other than A, C, G, T are observed, duplicates are observed, indels, monomorphic sites, allele mismatch between reference panel vs study data and SNP call rate of < 90%.

Third, the followings are checked at sample level:

1. For chromosomes 1 to 22 analyzed here, a chunk is excluded if one sample has a call rate < 50 % with only the complete chunks excluded and not samples.
2. Liftover is performed if the build of the input data and reference panel does not match.

Following the above steps, we applied the r2 threshold of 0.3 which removed >70% of poorly imputed SNPs at the cost of < 0.5% well imputed SNPs and minor allele frequency of 1%.

Supplementary figures 1-3 show the allele frequency correlation of arrays versus HRC reference panel.

**Imputation quality across the arrays**

After performing the QC steps described in the main manuscript under “Genotyping and imputation” section and supplemental information provided above about imputation procedure using the Michigan Imputation Server, 9,298,012 SNPs in the Illumina Array, and 11,081,999 SNPs in the PsychArray remained for analysis. In total, 9,008,825 of these SNPs were shared across the two arrays. We merged these shared SNPs with HapMap3 SNPs as described in the methods section under “polygenic risk score construction” for PRS construction and all downstream analyses. This left us with 943,020 high quality SNPs with imputation quality score of at least 0.96 across both arrays for PRS construction. Additional information is provided in supplementary table 3 below.

**Principal component analysis**

The principal component analysis (PCA) was performed using PLINK (4). We used the 1000 Genomes phase 3 data set populations as the background for our continental PCA analysis (5). Variants not on the coding strand from the 1000 Genomes Phase 3 dataset were first removed and only the variants with the minor allele frequency of more than 0.10 were retained. Next, all the other variants for each chromosome were pruned using the flag --indep 50 5 1.5. This left us of 249,512 variants on the 1000 Genomes Phase 3. High quality Imputed genotypes with imputation quality score of more than 0.99 were extracted from each of the three arrays and merged with the variants from the 1000 Genomes Phase 3 dataset to get a list of all the available SNPs across both platforms and 1000 Genomes Phase 3 for the continental PCA analysis. The total number of variants shared across both arrays and the 1000 Genomes Phase 3 was 164,307 variants. We then used –pca flag in PLINK V.1.9 to calculate the top 20 PCAs. After confirming that all the individuals in the sample are of European ancestry, a separate PCA was conducted on only the Irish subjects to capture the fine-scale population differences within the Irish population and these Irish specific PCAs were used as covariates in the analyses. Additionally, we note that only an unrelated subset of the ISHDSF sample were used for the initial PCA calculation – these values were then projected on the rest of the sample using the –pca-cluster-names flag. This ensured that only a subset of unrelated individuals was used for the initial PC calculation to avoid introducing possible bias/inflation in PC values due to relatedness of individuals. Our results indicate that while all individuals in the sample are of European ancestry (Supplementary Figures 1-2 below), to account for fine-scale population differences within the Irish population (Supplementary Figure 3), we also included the top 10 PCs as covariates in all the analysis.

**Covariates and accounting for possible batch effects**

Subjects used in this study were genotyped on 2 different arrays as shown in the supplementary table 4. These 2 arrays are the Illumina 610-Quad Array, and the Illumina Infinium PsychArray V.1.13. The bulk of the ISHDSF sample were genotyped on the illumine 610-Quad array by the Illumina. Additional ISHDSF subjects were genotyped on the PsychArray at Mount Sinai. A number of individuals for whom we already had genotypes available on the Illumina 610-Quad Array, were genotyped again on the PsychArray for quality control purposes. We compared and contrasted the genotypes as well polygenic risks constructed for these duplicate individuals and observed no significant differences, with high concordance rate. Additionally, the PCs on the Irish individuals showed no significant association with any of the genotyping batches or sites, indicating that there is no systematic differences or biases between these two arrays. However, to control for any other possible batch or site effects that escaped us, we included each of the platforms and genotyping sites as covariates in the models. Additionally, as described above, although the PCA shows that all the individuals analyzed in this study are of European ancestry, to account for fine-scale population structure of the Irish population, we also included the top 10 PCs as the covariates in our analyses.

**Separating negative and disorganized symptoms in schizophrenia**

The factor analysis of OPCRIT in our dataset differed slightly from other analyses. While different studies (as referenced in the manuscript) have shown that negative and disorganized symptoms sometimes load on to a single factor, this could potentially result in noisy interpretation of the results. Since negative/disorganized symptom dimension of OPCRIT showed a significant association with SCZ PRS in our study, we attempted to carry out a post-hoc analysis on this dimension by separating the symptoms into negative only and disorganized only symptoms. This step was taken to narrow down the association signals. Items included in the narrowed-down disorganized only dimension were bizarre behavior, catatonia, speech difficult to understand, positive formal thought disorder, and inappropriate affect. Items included in the narrowed-down negative only dimension were incoherent, negative formatl thought disorder, restricted affect, blunted affect, rapport difficult, information not credible, and deterioration from premorbid level of function. Items comprising the disorganized only dimension are also highlighted in the supplementary table 1.

Association analyses between SCZ PRS and these two narrowed-down dimensions showed significant association between SCZ PRS and both symptom groups (negative only *p* = 9.10 x 10 ^-3^, disorganized only *p* = 1.41 x 10^-5^). These results indicated that separating this dimension into separate negative and disorganized dimension did not result in full attenuation of the signals in one of the symptom groups, rather, both still showed significant association with SCZ PRS. However, we note that the significance level in disorganized only symptoms is higher than that observed with negative only symptoms, which is in agreement with a previous observation in a PRS analyses in the Molecular Genetics of Schizophrenia sample (6).

**Supplementary Tables:**

| **Factor** | **Item** | **Loading** |
| --- | --- | --- |
| **Negative/**  **Disorganized**  **Dimension** | 1. Bizarre behavior* | 0.538 |
|  | 2. Catatonia* | 0.544 |
|  | 3. Speech difficult to understand* | 0.623 |
|  | 4. Incoherent | 0.842 |
|  | 5. Positive format thought disorder* | 0.621 |
|  | 6. Negative formal thought disorder | 0.777 |
|  | 7. Restricted affect | 0.679 |
|  | 8. Blunted affect | 0.817 |
|  | 9. Inappropriate affect* | 0.6 |
|  | 10. Rapport difficult | 0.815 |
|  | 11. Information not credible | 0.813 |
|  | 12. Deterioration from premorbid level of function | 0.783 |
| **Delusions**  **Dimension** | 13. Persecutory delusions | 0.725 |
|  | 14. Well-organized delusions | 0.707 |
|  | 15. Delusions of influence | 0.709 |
|  | 16. Bizarre delusions | 0.86 |
|  | 17. Widespread Delusions | 0.806 |
|  | 18. Delusions of passivity | 0.944 |
|  | 19. Thought insertion | 0.898 |
|  | 20. Thought withdrawal | 0.707 |
|  | 21. Thought broadcast | 0.723 |
| **Hallucinations**  **Dimension** | 22. Delusions and hallucinations > 1week | 0.971 |
|  | 23. Persecutory/jealous delusions with hallucinations | 0.968 |
|  | 24. Third person auditory hallucinations | 0.734 |
|  | 25. Running commentary voices | 0.659 |
|  | 26. Abusive/accusatory/persecutory voices | 0.796 |
|  | 27. Other nonaffective auditory hallucinations | 0.568 |
|  | 28. Nonaffective hallucination in any modality | 0.986 |
| **Manic**  **Dimension** | 29. Affective symptoms predominate | 0.866 |
|  | 30. Grandiose delusions | 0.482 |
|  | 31. Elevated mood | 0.962 |
|  | 32. Irritable mood | 0.711 |
|  | 33. Excessive activity | 0.935 |
|  | 34. Reckless activity | 0.842 |
|  | 35. Pressured speech | 0.953 |
|  | 36. Increased self-esteem | 0.879 |
|  | 37. Thoughts racing | 0.94 |
|  | 38. Distractibility | 0.892 |
|  | 39. Reduced need for sleep | 0.886 |
| **Depressive**  **Dimension** | 40. Schizophrenia symptoms at the same time as affective symptoms | 0.934 |
|  | 41. Dysphoria | 0.968 |
|  | 42. Agitated activity | 0.615 |
|  | 43. Slowed activity | 0.759 |
|  | 44. Loss of energy/tiredness | 0.886 |
|  | 45. Loss of pleasure | 0.897 |
|  | 46. Poor concentration | 0.826 |
|  | 47. Excessive self-reproach | 0.865 |
|  | 48. Suicidal ideation | 0.724 |
|  | 49. Initial insomnia | 0.863 |
|  | 50. Early morning waking | 0.869 |
|  | 51. Excessive sleep | 0.523 |
|  | 52. Poor appetite | 0.904 |
|  | 53. Weight loss | 0.86 |
|  | 54. Increased appetite | 0.487 |
|  | 55. Weight gain | 0.489 |

**Supplementary Table 1:** 55 items used in the factor analysis of OPCRIT data in the ISHDSF sample. Factor loadings of OPCRIT items onto the 5 dimensions based on the factor analysis conducted by Fanous et al (7) are shown in column 3. Items highlighted with an asterisk for negative/disorganized dimension are the disorganized items.

| **Subjects with Operational Criteria Checklist for Psychotic Disorders (OPCRIT) Assessment** | |
| --- | --- |
| **Narrow (N=457)** | Schizophrenia N=261 |
|  | Schizophrenia Paranoid-type N = 60 |
|  | Schizophrenia Disorganized-type N = 62 |
|  | Schizophrenia Catatonic-type N = 11 |
|  | Schizophrenia Simple-type N = 7 |
|  | Poor-outcome schizoaffective disorder N = 56 |
|  |  |
| **Intermediate (N =68)** | Delusional disorder N = 4 |
|  | Psychotic disorder NOS N = 29 |
|  | Good-outcome schizoaffective disorder N = 28 |
|  | Schizophreniform N = 1 |
|  | Schizotypal personality disorder N = 6 |
|  |  |
| **Broad (N=14)** | Bipolar disorder = 8 |
|  | Major depression with psychotic features N = 5 |
|  | Paranoid personality disorder = 1 |

**Supplementary Table 2:** List of the diagnoses in narrow, intermediate, and broad spectrums with OPCRIT information available.

| **Subjects with Structured Interview for Schizotypy (SIS) Assessment** | |
| --- | --- |
| **Broad (N=13)** | Delusional disorder (N=2) |
|  | Avoidant personality disorder (N=9) |
|  | Schizoid personality disorder (N=2) |
|  |  |
| **Very Broad (N=137)** | Bipolar disorder (N =6) |
|  | Generalized anxiety disorder (N=22) |
|  | Major depressive disorder (N=77) |
|  | alcohol abuse + alcohol dependence (N=25) |
|  | Panic disorder (N=6) |
|  | Anorexia (N=1) |
|  |  |
| **Unaffected (N=172)** | Unaffected relatives of probands (N=172) |

**Supplementary Table 3:** List of the diagnoses in broad, and very broad spectrums with SIS information available. Note that SIS was also performed on unaffected relatives of patients in the families.

| **Array** | **N**  **ISHDSF** | **N**  **SNPs QC'd pre-imputation** | **N**  **Imputed SNPs QC'd** | **N**  **OPCRIT** | **N**  **SIS** |
| --- | --- | --- | --- | --- | --- |
| **Illumina 610-Quad Array** | 727 | 557,373 | 9,298,012 | 508 | 219 |
| **Infinium psychArray v.1.13** | 134 | 384,389 | 11,081,999 | 31 | 103 |

**Supplementary Table 4:** Description of the arrays used in this study. Number of individuals and pre/post imputation SNPs on each array are provided. Note that only individuals with symptom level information available were used in the current study. OPCRIT = Operational Criteria Checklist for Psychotic Disorders. SIS = Structured Interview for Schizotypy.

| **Array** | **Genotyped** | **Imputed** | **Mean r^2^ (SD)** |
| --- | --- | --- | --- |
| **Illumina 610-Quad** | 414,052 | 528,968 | 0.98 (0.035) |
| **Infinium PsychArray** | 338,265 | 604,755 | 0.96 (0.056) |

**Supplementary Table 5:** HapMap3 SNPs across the 2 arrays used for PRS construction. Mean imputation quality scores (SD) are provided on the 4^th^ column for the imputed SNPs.

| Base | Comparison | Ep | Minus2LL | Df | CFI | RMSEA | AIC | diffLL | Diffdf | P |
| --- | --- | --- | --- | --- | --- | --- | --- | --- | --- | --- |
| CFA3 factors |  | 39 | 4842.219 | 3066 | 0.823 | 0.096 | 4920.219 |  |  |  |
| CFA 3 factors | CFA 2 factors | 37 | 4809.670 | 3068 | 0.903 | 0.068 | 4883.670 | -32.54858 | 2 | 1 |

**Supplementary Table 6:** Model fit comparisons for the CFA on SIS data. CFA=Confirmatory factor analysis. Ep= estimated parameters. LL=Log-likelihood. AIC=Akaike’s information criterion.

**Supplementary Table 7:** Mixed-effects linear regression results for association between SCZ, BIP, and MDD PRS with OPCRIT and SIS symptom dimensions in psychotic and non-psychotic subjects in the ISHDSF sample. Adjusted *p*-values were generated using the Holm method in R. The analyses accounted for the family structure, sex, genotyping planforms, sites, and the top 10 PCs.

**Supplementary Table 8:** Mixed-effects quantile regression results for association between SCZ, BIP, and MDD PRS with OPCRIT and SIS symptom dimensions in psychotic and non-psychotic subjects in the ISHDSF sample. Adjusted *p*-values were generated using the Holm method in R. The analyses accounted for the family structure, sex, genotyping planforms, sites, and the top 10 PCs.

**Supplementary Figures:**

**Supplementary Figure 1:** Continental PCA plot for the samples genotyped on the Illumina array projected on the 1000 Genomes Phase 3 data. PC1 on the X-axis and PC2 on the Y-axis. Each color represents one of the ancestries. Black color represents the Irish sample.

**Supplementary Figure 2:** Continental PCA plot for the samples genotyped on the psychArray projected on the 1000 Genomes Phase 3 data. PC1 on the X-axis and PC2 on the Y-axis. Each color represents one of the ancestries. Black color represents the Irish sample.

**Supplementary Figure 3:** Fine-scale PCA analysis of the Irish population. The fine-scale population structure captured for PC1 on the X-axis and PC2 on the Y-axis, closely resembles the population structure of the British Isles. The top 10 PCs were used as covariates in the subsequent analyses. Blue color represents subjects from the ISHDSF sample genotyped on Illumina array. Black color represents subjects from the ISHDSF sample genotyped on the psychChip. Green color represents genotyped individuals from the Irish Biobank (8) super-imposed on ISHDSF sample for better visualization of the population structure of Ireland.


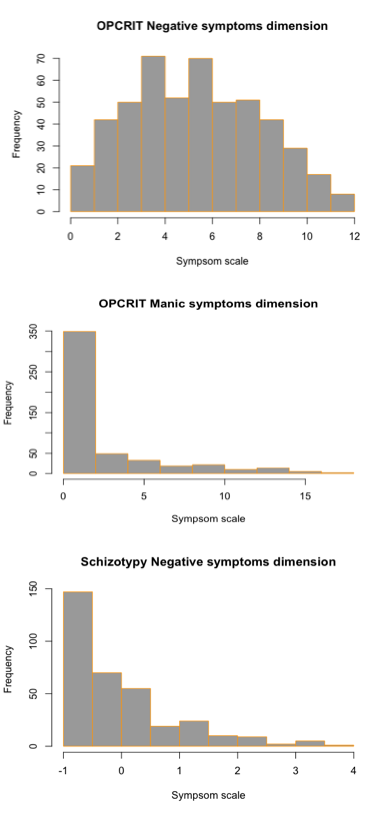


**Supplementary Figure 4:** Distribution of the symptom scores in the three symptom dimensions that show significant association with one of the polygenic risks. Note that OPCRIT negative symptom dimension is normally distributed, whereas manic OPCRIT symptom dimension and negative schizotypy symptom dimension are skewed with floor effect. Quantile regression analyses were conducted to investigate the association at different locations in the distribution.

**Supplementary Figure 5:** The quantile regression estimates and ordinary least squares (OLS) estimates visualized on each plot. **A.** OPCRIT negative symptoms. **B.** OPCRIT manic symptoms. **C.** SIS schizotypy negative symptoms. The quantile estimates are visualized at 0.1 increments as black lines with the corresponding confidence intervals shown in gray. The three quantiles are depicted as three dots corresponding to Q1 (0.25), Q2 (0.5), and Q3 (0.75) of symptom score distributions. In the background, OLS regression estimate is shown as a red line, with the dotted red lines depicting the confidence intervals. Note that due to the fairly normal distribution of OPCRIT negative symptoms, all three quantiles fell within the confidence intervals for OLS, whereas OPCRIT manic symptoms and SIS schizotypy negative symptoms show a different pattern.

**References**

1. Kendler KS, O’Neill FA, Burke J, Murphy B, Duke F, Straub RE, et al. Irish study of high-density schizophrenia families: Field methods and power to detect linkage. Am J Med Genet - Semin Med Genet. 1996;67(2):179–90.

2. Kendler KS, McGuire M, Gruenberg AM, Spellman M, O’Hare A, Walsh D. The Roscommon Family Study: II. The Risk of Nonschizophrenic Nonaffective Psychoses in Relatives. Arch Gen Psychiatry. 1993;50(8):645–52.

3. Das S, Forer L, Schönherr S, Sidore C, Locke AE, Kwong A, et al. Next-generation genotype imputation service and methods. Nat Genet. 2016;48(10):1284–7.

4. Purcell S, Neale B, Todd-Brown K, Thomas L, Ferreira MAR, Bender D, et al. PLINK: A Tool Set for Whole-Genome Association and Population-Based Linkage Analyses. Am J Hum Genet. 2007;81(3):559–75.

5. Auton A, Abecasis GR, Altshuler DM, Durbin RM, Bentley DR, Chakravarti A, et al. A global reference for human genetic variation. Nature. 2015;526(7571):68–74.

6. Fanous AH, Zhou B, Aggen SH, Bergen SE, Amdur RL, Duan J, et al. Genome-wide association study of clinical dimensions of schizophrenia: Polygenic effect on disorganized symptoms. Am J Psychiatry. 2012;169(12):1309–17.

7. Fanous AH, Van Den Oord EJ, Riley BP, Aggen SH, Neale MC, O’Neill FA, et al. Relationship between a high-risk haplotype in the DTNBP1 (dysbindin) gene and clinical features of schizophrenia. Am J Psychiatry. 2005;162(10):1824–32.

8. Donnelly P, Barroso I, Blackwell JM, Bramon E, Brown MA, Casas JP, et al. Genome-wide association study implicates HLA-C*01:02 as a risk factor at the major histocompatibility complex locus in schizophrenia. Biol Psychiatry. 2012;72(8):620–8.
